# The Proportion of Soil-Borne Fungal Pathogens Increases with Elevated Organic Carbon in Agricultural Soils

**DOI:** 10.1128/msystems.01337-21

**Published:** 2022-03-21

**Authors:** Shuai Du, Pankaj Trivedi, Zhong Wei, Jiao Feng, Hang-Wei Hu, Li Bi, Qiaoyun Huang, Yu-Rong Liu

**Affiliations:** a State Key Laboratory of Agricultural Microbiology, Huazhong Agricultural University, Wuhan, China; b College of Resources and Environment, Huazhong Agricultural University, Wuhan, China; c Microbiome Network and Department of Agricultural Biology, Colorado State University, Fort Collins, Colorado, USA; d Jiangsu Provincial Key Lab for Organic Solid Waste Utilization, Jiangsu Collaborative Innovation Center for Solid Organic Waste Resource Utilization, National Engineering Research Center for Organic-based Fertilizers, Nanjing Agricultural University, Nanjing, China; e School of Agriculture and Food, Faculty of Veterinary and Agricultural Sciences, The University of Melbourne, Parkville, Victoria, Australia; University of Waterloo

**Keywords:** agricultural soil, fertilization management, fungal phytopathogen, organic carbon, soil health

## Abstract

Soil-borne fungal phytopathogens are important threats to soil and crop health. However, their community composition and environmental determinants remain unclear. Here, we explored the effects of agricultural fertilization regime (i.e., organic material application) on soil fungal phytopathogens, using data sets from a combination of field survey and long-term experiment. We found that soil organic carbon was the key factor that affected the diversity and relative abundance of fungal phytopathogens in agricultural soils. The dominant genera of phytopathogens including *Monographella* was also strongly associated with soil organic carbon. In addition, the elevated soil organic carbon enhanced the node proportion of phytopathogens and the positive interactions within the fungal community in the network. Results of the long-term experiment revealed that applications of crop straw and fresh livestock manure significantly increased the proportion of phytopathogens, which were associated with the elevated soil organic carbon. This work offers new insights into the occurrence and environmental factors of fungal phytopathogens in agricultural soils, which are fundamental to control their impacts on the soil and crop systems.

**IMPORTANCE** Fungal phytopathogens are important threats to soil and crop health, but their community composition and environmental determinants remain unclear. We found that soil organic carbon is the key factor of the prevalence of fungal phytopathogens through a field survey, which is also supported by our long-term (6-year) experiment showing the applications of crop straw and fresh livestock manure significantly increased the proportion of fungal phytopathogens. These findings advance our understanding of the occurrence and environmental drivers of soil-borne fungal phytopathogens under agricultural fertilization regime and have important implications for the control of soil-borne pathogens.

## INTRODUCTION

Soil health is a global concern linking tightly to crop production and food security, but it has been threatened by various soil-borne phytopathogens worldwide (1–5). Soil-borne phytopathogens can infect a wide range of agricultural and economic crops (6, 7), and have caused up to 78% loss in the productivity of cereal crops, vegetables, and fruits (8). Many of the most aggressive phytopathogens are soil-borne fungi, which have been predicted to increase by up to 3-fold by 2050 globally (9). Mounting studies have focused on the occurrence and causes of certain fungal pathogens that prevail in soil–plant systems and provided essential information on how soil pathogens such as Fusarium graminearum, Magnaporthe oryzae, and Bipolaris sorokiniana infect plants including maize, wheat, and rice (6, 10–12). However, the prevalence and diversity of the whole community of fungal phytopathogens in agricultural soils is still unclear, although they are the potential causes of plant diseases (13–15). Therefore, deciphering the community composition of soil fungal pathogens and their determinants is crucial to protect crop health from diseases.

Soil microbial communities are susceptible to agricultural management practices for sustaining crop productivity. A large body of literature has demonstrated the effects of fertilization regime on the soil fungal community (16–18). Importantly, fertilization regimes such as organic material application (e.g., crop straw or livestock manure) are a key regulator of soil fungal community through introducing abundant nutrients into soil (19, 20). Also, organic material application can affect soil properties such as pH and quality and quantity of organic matter that are associated with fungal community composition (21–24). Fungal communities can be categorized into different trophic guilds, including pathotrophs, saprotrophs, and symbiotrophs, and they may have different demands for nutrients (25–27). For example, it has been reported that nitrogen and phosphorus addition increased the proportion of soil-borne fungal pathotrophs and decreased symbiotrophs, but had minimal effects on saprotrophs (28). However, it is still poorly understood how organic material application may affect different fungal trophic communities, especially pathogens in agricultural soils.

The interactions within fungal communities are associated with ecological functions such as nutrient cycling. Pathogenic fungi can interact with other fungi, competing for nutrients and space in soils. For example, pathogenic Fusarium oxysporum competes for carbon, nitrogen, and iron with nonpathogenic Fusarium and Trichoderma species (29). Mycorrhizal fungi are reported to compete for space with fungal pathogens such as Plasmodiophora brassicae and Plasmopara halstedii because of their tight associations with plant roots (30). However, the influence of soil organic matter on the interactions between different fungal trophic guilds is still not clear. Consequently, understanding microbial interactions between soil fungal pathogens and other microbial taxa is particularly important for possible control of them.

Herein, we aimed to evaluate how organic material application influences the diversity, relative abundance, and community composition of soil-borne fungal phytopathogens, and identify the key environmental predictors. We hypothesized that organic material application facilitated the occurrence of fungal phytopathogens by influencing soil chemical properties including organic carbon. To achieve this aim, we conducted a field survey of agricultural soils in the major grain-producing areas of China, combining a long-term field experiment including multiple organic fertilizer applications. We used fungal internal transcribed spacer (ITS) amplicon sequencing to characterize the diversity, relative abundance, and community composition of soil-borne fungal phytopathogens, and then identified key regulators associated with these pathogens in agricultural soils. These results were expected to move toward our understanding of soil-borne fungal phytopathogens in agricultural systems.

## RESULTS

### The diversity, relative abundance, and environmental predictors of fungal trophic guilds in agricultural soils.

**FIG 1:**
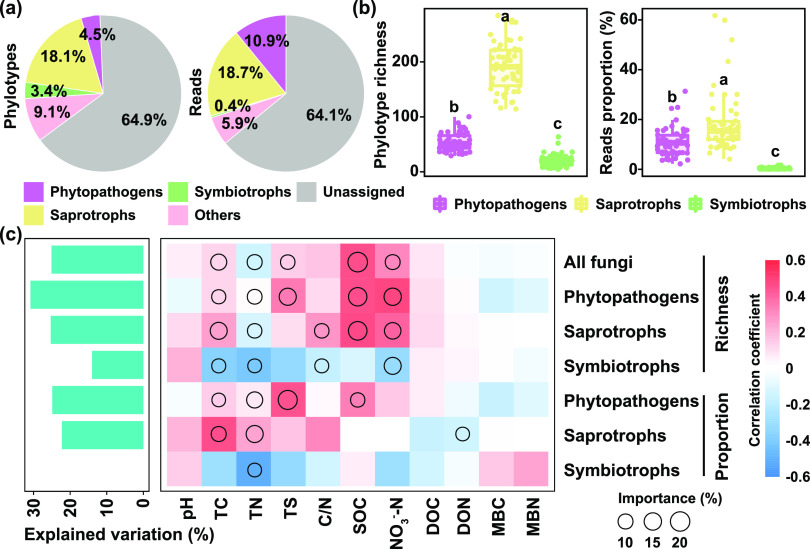
The proportion of phylotypes and reads of different fungal trophic modes, and the effects of soil properties on them in agricultural soils. (a) The proportion of phylotypes and reads of different fungal trophic modes. (b) Phylotype richness and reads proportion of three fungal trophic modes. One-way ANOVA indicates the difference between fungal trophic modes, and different letters indicate significant difference (*P < *0.05) based on Duncan test. (c) Soil properties associated with the diversity and relative abundance of all fungi and three trophic modes evaluated by Spearman correlation and best random forest model. Colors represent Spearman correlations. Circle sizes represent the variable's importance (that is, decrease in the prediction accuracy). TC, total carbon; TN, total nitrogen; TS, total sulfur; C/N, carbon–nitrogen ratio; SOC, soil organic carbon; DOC, dissolved organic carbon; DON, dissolved organic nitrogen; MBC, microbial biomass carbon; MBN, microbial biomass nitrogen.

We identified 276, 1102, and 204 phylotypes (OTUs) classified as potential phytopathogens, saprotrophs, and symbiotrophs out of all the 6,075 fungal phylotypes. The potential phytopathogens, saprotrophs, and symbiotrophs accounted for 4.5%, 18.1%, and 3.4% of all fungal phylotypes, and accounted for 10.9%, 18.7%, and 0.4% of all fungal reads, respectively (Fig. 1a). The phylotype richness and reads proportion of saprotrophs were significantly higher than those of phytopathogens and symbiotrophs (*P < *0.01, ANOVA; Fig. 1b). A complete list of the potential soil-borne fungal phytopathogens, saprotrophs, and symbiotrophs included in the field survey can be found in Table S1 in the supplemental material.

We explored the effects of soil properties on the diversity (phylotype richness) and relative abundance (reads proportion) of all fungi and three trophic guilds (i.e., phytopathogens, saprotrophs, and symbiotrophs) in agricultural soils. The richness of all fungi, phytopathogens, and saprotrophs were positively correlated with soil organic carbon (SOC), total carbon (TC), and NO_3_^−^-N (Fig. 1c). The proportion of phytopathogens was positively correlated with SOC, TC, and total sulfur (TS), while no significant correlations were observed between these soil properties and other two trophic guilds (Fig. 1c). The result of random forest showed that phytopathogens had the highest explained variation by soil properties. A complete list of soil chemical properties included in the field survey can be found in Table S2. The community structure of all fungi and three trophic guilds were also associated with SOC (Fig. S1).

### The relative abundance of fungal phytopathogenic genera and their correlations with soil properties.

**FIG 2:**
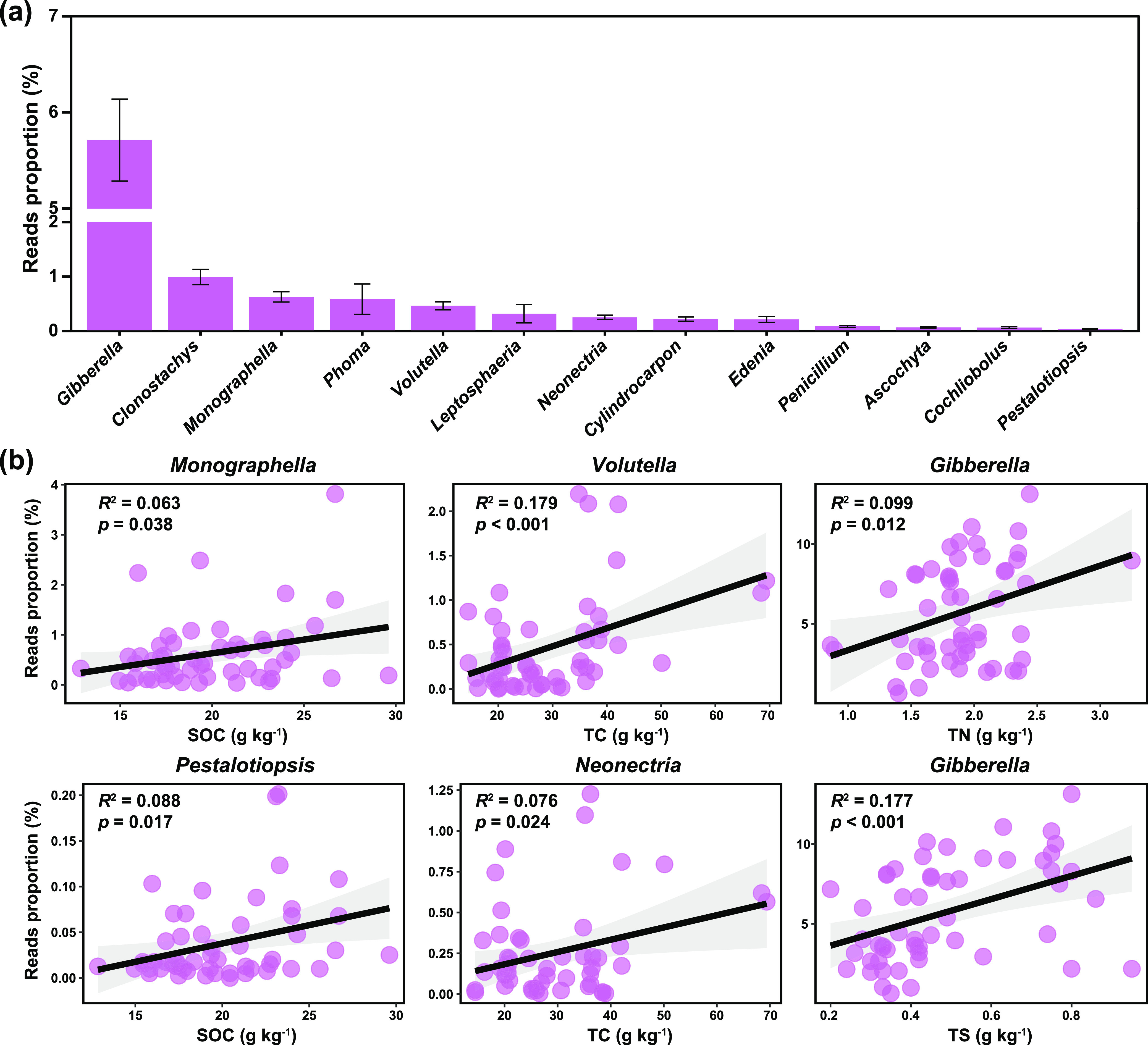
The dominant genera of fungal phytopathogens and the effects of soil properties on them in agricultural soils. (a) The dominant genera (the relative abundance more than 0.01%) of fungal phytopathogens in agricultural soils. (b) The regressive relationships between soil properties (i.e., SOC, TC, TN, TS) and the relative abundance of all fungi, phytopathogens, saprotrophs, and symbiotrophs. SOC, soil organic carbon; TC, total carbon; TN, total nitrogen; TS, total sulfur.

We identified 13 dominant genera of fungal phytopathogens (the relative abundance more than 0.01%) such as *Gibberella*, *Clonostachys*, *Monographella*, *Phoma*, *Volutella*, *Leptosphaeria*, and *Neonectria*, accounting for 88.1% of all phytopathogens in abundance (Fig. 2a). Among them, *Gibberella* was the most abundant phytopathogenic genus, with the relative abundance of 5.7%. The pathogenic genera *Monographella* and *Pestalotiopsis* were positively correlated with SOC, while *Volutella* and *Neonectria* were positively correlated with TC, and the genus *Gibberella* was positively correlated with total nitrogen (TN) and TS (*P < *0.05, Fig. 2b).

### Network interactions between trophic guilds within fungal community.

**FIG 3:**
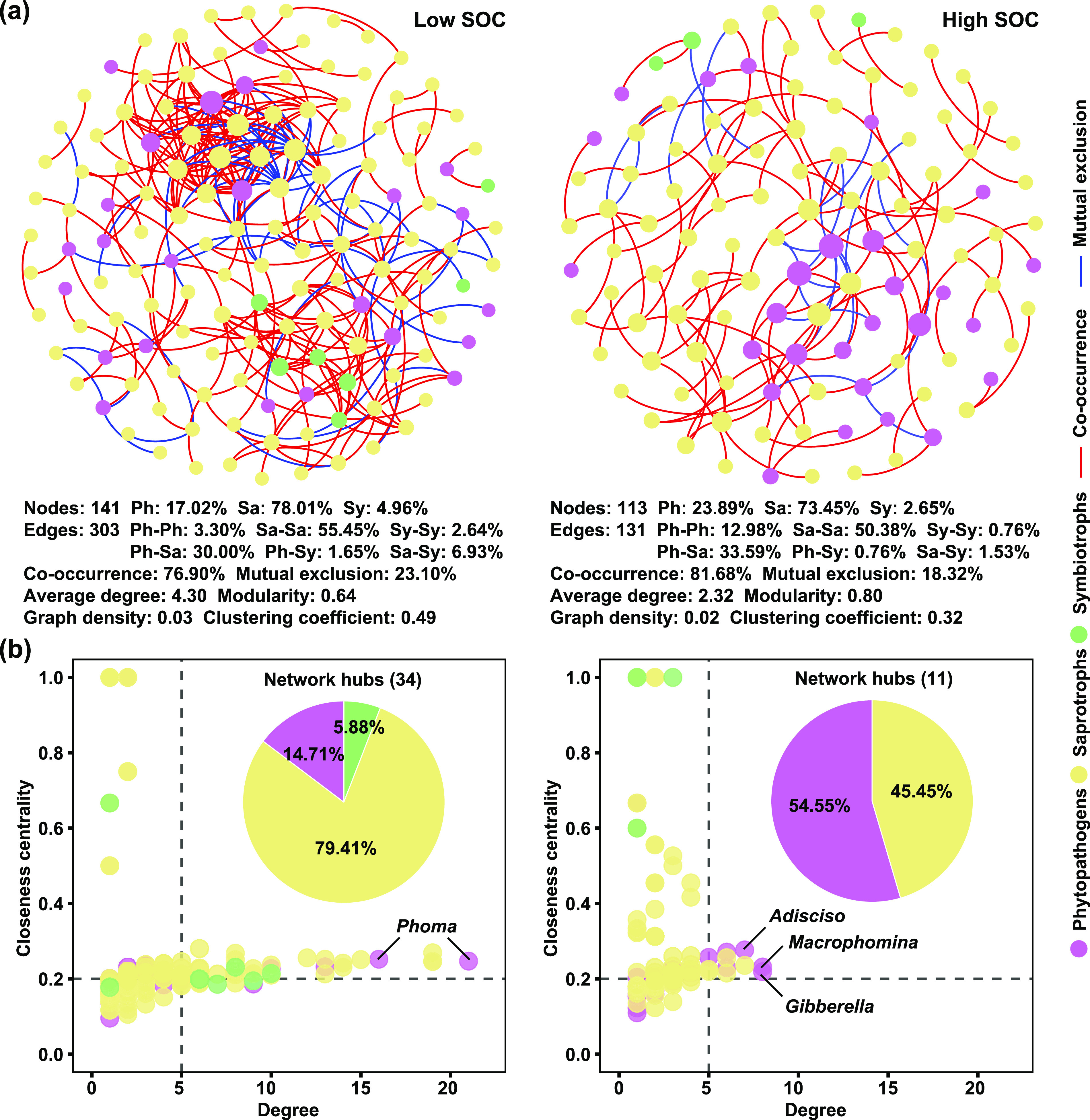
Networks visualizing the co-occurrence patterns between phytopathogens, saprotrophs, and symbiotrophs. (a) The co-occurrence networks between phytopathogens, saprotrophs, and symbiotrophs in low and high SOC groups. The node size is proportional to the degree of taxa, and the nodes filled in different colors are different trophic modes of fungi. The edges are colored according to interaction types, positive correlations are colored in red, and negative correlations are colored in blue. Ph, phytopathogens; Sa, saprotrophs; Sy, symbiotrophs. (b) The distribution patterns of the hub nodes in low and high SOC groups. The hub nodes were defined as degree > 5, and closeness centrality > 0.2.

The co-occurrence network was used to explore the potential interactions between different fungal guilds in agricultural soils. The soil samples were separated into two groups based on the median value of SOC content (∼20 g kg^−1^) in all samples. The node proportion of phytopathogens in the high SOC group (> 20 g kg^−1^) was 1.40 times that in the low SOC group (< 20 g kg^−1^) (Fig. 3a). The proportion of positive correlated edges increased 4.78% from low to high SOC groups, and the proportion of interactions within phytopathogens increased 9.68%, but the average degree decreased 1.98 (Fig. 3a). The fungal hub nodes were 34 and 11 in the two networks, with an increasing proportion of phytopathogenic hub nodes from 14.71% to 54.55% (Fig. 3b). The phytopathogenic hub nodes were represented by *Phoma* in the low SOC group, and *Gibberella*, *Macrophomina*, and *Adisciso* in the high SOC group (Fig. 3b).

### Effects of long-term organic material applications on soil fungal phytopathogens.

To experimentally corroborate the observations in the field survey, we analyzed the effects of fertilization regime on soil fungal phytopathogens from a 6-year field experiment. We focused on the effects of application of organic materials on fungal phytopathogens, which can be an important way introducing diverse pathogens. We identified 33 phylotypes classified as potential phytopathogens out of all the 815 fungal phylotypes in all the treatments. A complete list of the potential soil-borne fungal phytopathogens, saprotrophs, and symbiotrophs included in the experiment can be found in Table S3.

**FIG 4:**
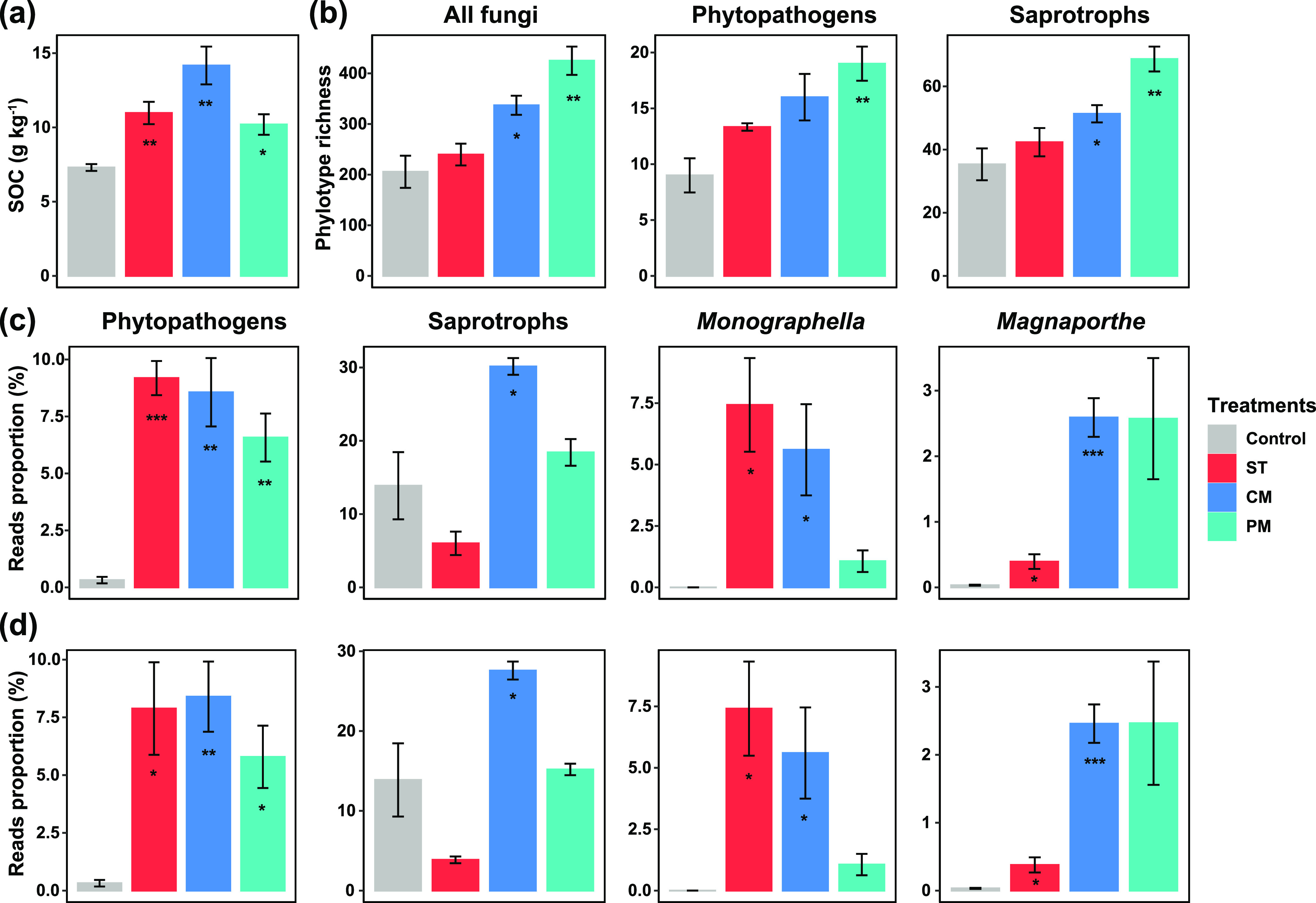
Soil properties, fungal trophic guilds, and the dominant genera of phytopathogens in the long-term experiment. (a) Comparison of soil organic carbon in different fertilization treatments. (b) Comparison of the diversity of all fungi, phytopathogens, and saprotrophs in different fertilization treatments. (c) Comparison of the relative abundance of phytopathogens, saprotrophs, and the dominant genera of phytopathogens in different fertilization treatments. (d) Comparison of the relative abundance of shared phytopathogens, saprotrophs, and the dominant genera of phytopathogens in the control and fertilization treatments. Columns with asterisks in the treatments are significantly different from the control (*. *P < *0.05; **, *P < *0.01; ***, *P < *0.001). ST, crop wheat straw; CM, fresh cow manure; PM, fresh pig manure.

Long-term applications of wheat straw and livestock manure (i.e., cattle and pig manure) significantly enhanced SOC content (*P < *0.05; Fig. 4a), while other soil properties had different responses to different types of organic material applications (Fig. S2). The applications of pig manure significantly enhanced the richness of all fungi, phytopathogens, and saprotrophs (*P < *0.05, Fig. 4b). The relative abundance of phytopathogens was strongly increased in the treatments of straw, cattle, and pig manure from that of the control (*P < *0.05; Fig. 4c), while the relative abundance of saprotrophs was only increased in the treatment of cattle manure, and both the richness and the proportion of symbiotrophs were not different between the treatments and the control (*P > *0.05; Fig. S3a and b). In particular, the relative abundance of the dominant genera of phytopathogens such as *Monographella* and *Magnaporthe* was significantly increased in the treatments of straw and cattle manure (*P < *0.05; Fig. 4c). The application of pig manure significantly increased the proportion of phytopathogenic genera *Penicillium*, *Devriesia*, and *Pestalotiopsis* (*P < *0.05; Fig. S3c). We further found that the relative abundance of shared phytopathogens and dominant genera were significantly higher in the treatments of straw and cattle manure than that of the control (*P < *0.05; Fig. 4d), suggesting that the applications of organic materials increased the relative abundance of soil indigenous phytopathogens. The three fertilization treatments also altered the community structure of all fungi and three trophic guilds (*P < *0.05, PERMANOVA; Fig. S4).

**FIG 5:**
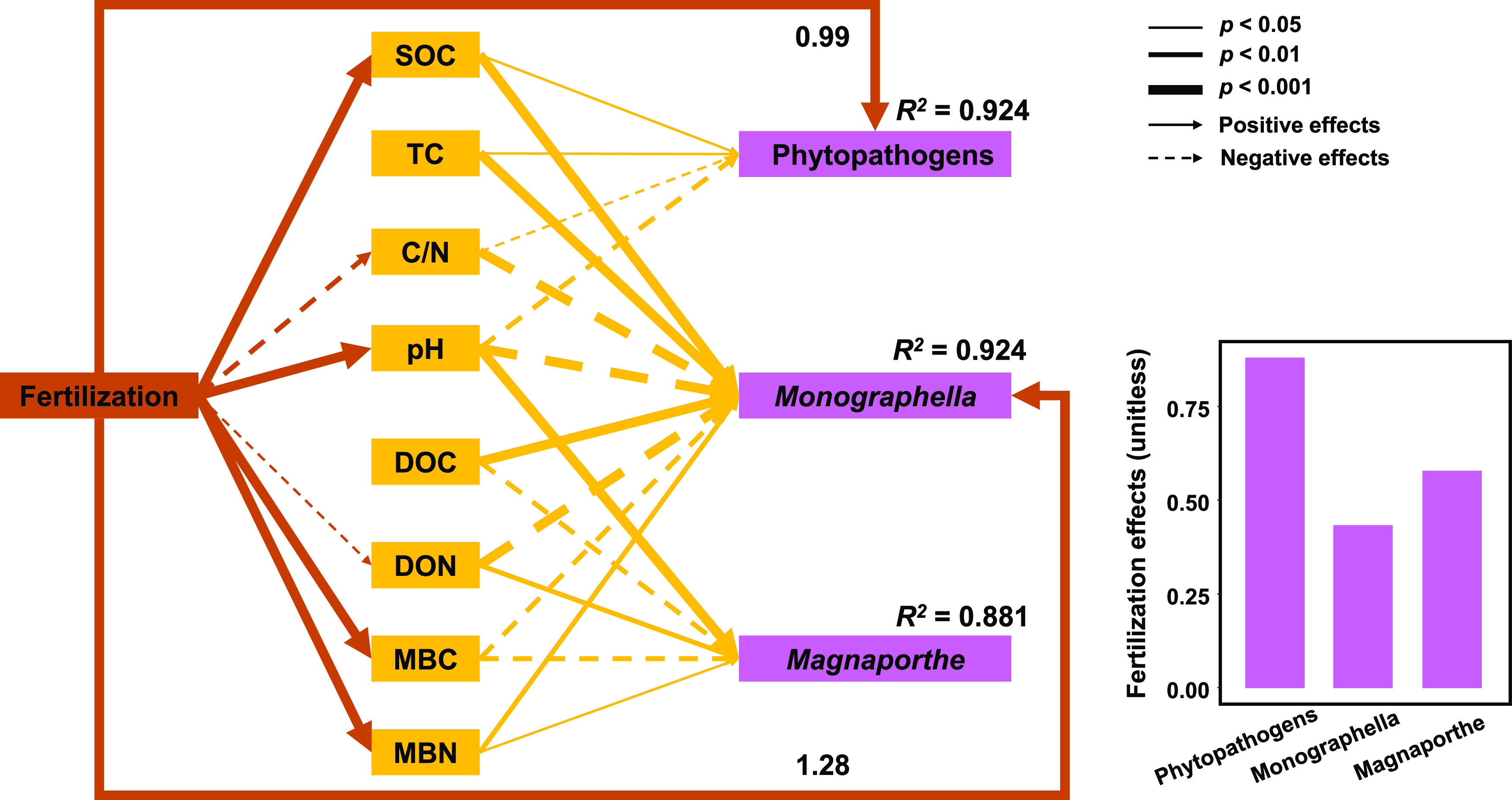
The effects of fertilization treatments on soil properties and phytopathogens in the long-term experiment. Path analysis assesses the direct and indirect associations of fertilization treatments and soil properties with the relative abundance of fungal phytopathogens and its two dominant genera. The standardized total effects (sum of the direct and indirect effects) of fertilization treatments are calculated on the relative abundance of fungal phytopathogens. The models fit the data well, as suggested by *χ*^2^ = 6.078, *P = *0.415, df = 6, GFI = 0.917, AIC = 104.078, and RMSEA = 0.034 (for phytopathogens); *χ*^2^ = 0.836, *P = *0.658, df = 2, GFI = 0.986, AIC = 106.836, and RMSEA = 0 (for *Monographella*); *χ*^2^ = 8.992, *P = *0.174, df = 6, GFI = 0.892, AIC = 106.992, and RMSEA = 0.213 (for *Magnaporthe*), respectively. The solid line represents the positive effects and the dashed line represents the negative effects. Only significant path coefficients are displayed, with the significance levels indicated: *, *P < *0.05; **, *P < *0.01; ***, *P < *0.001. The width of arrows is proportional to the significance levels. SOC, soil organic carbon; TC, total carbon; C/N, carbon nitrogen ratio; DOC, dissolved organic carbon; DON, dissolved organic nitrogen; MBC, microbial biomass carbon; MBN, microbial biomass nitrogen.

The results of path analysis revealed that the application of organic materials directly influenced the proportions of phytopathogens and the dominant genera including *Monographella* and *Magnaporthe* (Fig. 5). In addition, fertilization treatments also had indirect effects on phytopathogens through the associations with soil properties including SOC, C/N, and pH (Fig. 5). After accounting for the standardized total effects of fertilization treatments, the results showed that the application of organic materials had strong effects on phytopathogens, followed by *Magnaporthe* and *Monographella* (Fig. 5). A complete list of soil chemical properties included in the experiment can be found in Table S4.

## DISCUSSION

Understanding the occurrence of fungal pathogens in agricultural soils is an essential to control plant diseases in crop production. However, we lack understandings of the distribution patterns and influence factors of fungal phytopathogens in agricultural soils. Here, we demonstrate that organic material application is a key regulator of soil fungal community of phytopathogens. Crop straw and fresh manure significantly aggravate the prevalence of soil-borne pathogens by increasing the content of soil organic carbon. These results provide new insights into the ecology of these phytopathogens and potential mitigation of soil-borne pathogens in response to intensified agriculture.

In this study, the proportion of phytopathogenic reads was far higher than the proportion of phytopathogenic phylotypes, while the proportion of saprotrophic reads was similar to the proportion of saprotrophic phylotypes. This result implied that phytopathogenic fungi might exert more important ecological effects than saprotrophic fungi, although the relative abundance of phytopathogens was lower than saprotrophs. Soil with high abundance of phytopathogens could be prone to result in plant diseases (31, 32). The positive correlation between soil organic carbon content and the proportion of phytopathogens within the whole fungal community suggests that soil organic matters can stimulate phytopathogens. Our results implicate that organic carbon has larger effects on phytopathogenic fungi than saprotrophic and symbiotrophic fungi in agricultural soils. It has been documented that phytopathogens, saprotrophs, and symbiotrophs were mainly influenced by soil properties, climate, and plant community, respectively, based on the global distribution patterns of fungal guilds (28). Fungal phytopathogens could acquire nutrients to grow and reproduce with different strategies, and are affected by nutrient availability (33). It has been reported that pathogens could deactivate plant defenses from preventing nutrient acquisition (34). The infection of pathogenic fungi like the filamentous fungus Aspergillus fumigatus includes key metabolic pathways, with amino acids served as nutrients during infection (33).

Our study identified several dominant genera of fungal phytopathogens in agricultural soils, which had the potential to cause plant diseases. For example, the genus Gibberella (also considered as Fusarium) is ubiquitous in agricultural soils, and most species of this genus (such as Fusarium oxysporum, Fusarium fujikuroi, and Fusarium graminearum) can affect economically important crops (including cereal crops and tomato) (11, 35). Another genus, Monographella, has been reported to be pathogenic to crops including spring barley (36). Other genera of phytopathogens such as Clonostachys, Leptosphaeria, Phoma, and Colletotrichum also commonly occur in agricultural soils (37–40), posing serious threats to soil and crop health.

Microbial interactions also play roles in regulating soil fungal phytopathogens. The potential cooperation and competition between microbial taxa are often shown by positive and negative connections between two nodes in a co-occurrence network (41). Here, higher proportion of phytopathogens and more interactions within phytopathogens were found in the network of high SOC group than that of low SOC group, suggesting that the prevalence of phytopathogens was enhanced in higher SOC content. The higher proportion of positive connections was found in the network of high SOC group, suggesting more cooperation between fungal taxa in the condition of higher SOC content. It was reported that pathogens could be more prone to invade a microbial community with more cooperation than competition, because microbial community with more cooperation could produce more public goods, which might benefit pathogens (42). Similarly, the network of microbial community had more positive connections in diseased soils than in healthy soils (31). The competitions often occur between pathogens and beneficial microorganisms of the plant (30, 43).

Previous studies revealed that fungal phytopathogens were driven by climate conditions including warming and precipitation in the ecosystems with less human disturbance (e.g., forest and grassland) (9, 44). While in agroecosystems, fungal phytopathogens might be mainly affected by agricultural managements. Our results suggest that fertilization management regime is a vital factor influencing fungal phytopathogens in agricultural soils. It has been reported that nitrogen and phosphorus fertilization consistently favor pathogenic fungi in grassland soils (28), while organic fertilizers also promote pathogenic fungi. The applications of crop straw and livestock manure could improve soil nutrition condition for microbial communities (45, 46). In this study, the higher proportions of fungal phytopathogens and the genera *Monographella* and *Magnoporthe* in the fertilization treatments suggest that nutrients could influence the prevalence of soil-borne pathogens. This could be attributed to the increasing soil organic carbon after the applications of crop straw and livestock manure. Crop straw return provided suitable conditions for pathogens to grow, propagate, and accumulate, thus resulting in soil-borne diseases (47–50). For example, it has been reported that maize straw return enhanced the abundances of two fungal phytopathogens i.e., F. graminearum and F. moniliforme (51).

The increased diversity and proportion of fungal phytopathogens could also be explained the applications of organic materials introducing abundant and diverse potential pathogens (52–54). For example, maize and wheat straw have been reported to be potential sources harboring fungal phytopathogens (e.g., Fusarium moniliforme, Fusarium proliferatum, Fusarium graminearum, and Fusarium subglutinans) (51, 55, 56). It has been estimated that the yields of crop straw and livestock fresh manure are 9 × 10^8^ tons and 3.8 × 10^9^ tons every year in China, respectively, with approximately 56% of crop straw used as fertilizers (data from http://www.cnr.cn/). The returned crop straw and livestock fresh manure may thus bring considerable amounts of pathogens to agricultural soils. Our results suggest that organic soil matter is an important predictor of fungal phytopathogens, and the application of organic materials is a key cause of the increased fungal phytopathogens in agricultural soils, which is pivotal to control plant diseases via regulating fertilization managements.

### Conclusions.

Taken together, our work provides novel insights into the prevalence and diversity of fungal phytopathogens in agricultural soils, which are the major causes of soil-borne diseases jeopardizing crop health worldwide. Our study found that the relative abundance of phytopathogens was positively correlated with organic soil carbon. We highlight that the application of organic materials is a vital factor influencing the prevalence of soil-borne fungal phytopathogens. These findings advance our understanding of the occurrence and sensitivities of soil-borne fungal phytopathogens to environmental changes, especially resulting from intensified agriculture where crop straw returning and fresh manure fertilization have become prevalent management practices. Our work suggests that caution should be taken when applying crop straw and fresh livestock manure to agricultural soils for enhancing fertility.

## MATERIALS AND METHODS

### Field survey of soil-borne fungal phytopathogens.

The field survey was conducted in major grain-producing areas in southwest China (Fig. S5) (57). Soil samples were collected from 18 locations covering maize planting fields in August 2016 before harvesting. Three replicated composite soil (0–15 cm depth) samples were collected at each location; thus, 54 total samples were obtained. Spatial information including longitude and latitude were recorded while sampling. Soil samples were homogenized and sieved (2 mm mesh) on arrival at the laboratory, and subsequently divided into two subsamples. One sub-ample was immediately stored at −20°C for molecular analyses, and the other subsample was stored at 4°C for chemical analyses. Soil chemical properties were measured according to the traditional methods (58). Briefly, soil pH was measured on a fresh soil-to-water ratio of 1:2.5 using a Delta pH meter. TC, TN, and TS were determined on a LECO TureMac Macro CN analyzer (LECO, St. Joseph, MI, USA). Carbon–nitrogen ratio (C/N) was calculated as the quotient between TC and TN. Soil organic carbon (SOC) was measured using the K_2_CrO_7_ oxidation titration method. Soil inorganic nitrogen (NH_4_^+^-N and NO_3_^−^-N) were measured using a SAN++ Continuous Flow Analyser (Skalar, Breda, Netherlands). Dissolved organic carbon (DOC) and total dissolved nitrogen (TDN) were determined with a Shimadzu TOC-TN analyzer (Shimadzu Corp., Kyoto, Japan). Dissolved organic nitrogen (DON) was calculated as the difference between the TDN reading and the combined NH_4_^+^-N and NO_3_^−^-N reading. Microbial biomass carbon (MBC) and nitrogen (MBN) were determined using the fumigation-extraction method.

### Long-term experiment of organic material applications.

The 6-year fertilization experiment was conducted with multiple organic material applications in east China (Linquan in Anhui Province) (59). The experimental plots were randomly arranged and subject to wheat–maize rotation, including four treatments with three replicate plots for each: (i) control; (ii) preceding wheat straw (ST); (iii) fresh cow manure (CM); (iv) fresh pig manure (PM). The NPK fertilizer comprised urea (300 kg N ha^−1^ y^−1^), superphosphate (120 kg P_2_O_5_ ha^−1^ y^−1^), and potassium chloride (100 kg K_2_O ha^−1^ y^−1^) for each treatment. For (iii) and (iv), 50% NPK fertilizers and 6,000 kg fresh manure ha^−1^ y^−1^ were applied. Soil samples (0–15 cm) from each plot were collected after the harvest of wheat and were homogenized and sieved (2 mm mesh) on arrival at the laboratory, followed by dividing into two subsamples and treated as described above. Soil properties including pH, TC, C/N, SOC, DOC, DON, MBC, and MBN were measured as described above.

### Soil DNA extraction and high-throughput sequencing.

The genomic DNA was extracted from 0.30 g of soil using the DNeasy PowerSoil Kit (Qiagen GmbH, Germany) according to the manufacturer’s instructions. The quality and concentration of extracted DNA were checked using the NanoDrop ND-2000c UV-Vis spectrophotometer (NanoDrop Technologies, Wilmington, DE, USA). Fungal communities from the field survey and the long-term experiment were determined by sequencing fungal ITS region with the primer pairs ITS1F (CTTGGTCATTTAGAGGAAGTAA) and 2043R (GCTGCGTTCTTCATCGATGC) on the Illumina MiSeq platform (2 × 250 PE) (Illumina Inc., San Diego, USA). Bioinformatic processing was performed using a combination of QIIME (60) and USEARCH (61). Then, UPARSE was employed to filter chimera, and the operational taxonomic unit (OTU or phylotype) was picked at 97% sequence similarity. Phylotype identification was obtained against the SILVA rRNA gene database (62). The relative abundance (%) of each phylotype was calculated from the resulting OTU (phylotype) table. Trophic guilds including phytopathogens, saprotrophs, and symbiotrophs of fungal communities were predicted by the FUNGuild database (10) (http://www.funguild.org/query.php?qText=&qDB=funguild_db&qField=taxon; accessed August 2020), and classified as probable and highly probable phylotypes (excluding possible phylotypes). The relative abundances of soil-borne fungal phytopathogens, saprotrophs, and symbiotrophs were calculated using rarefied ITS OTU tables in each sample of field survey or long-term experiment, as the sum of ITS reads of fungal phytopathogens (or saprotrophs, symbiotrophs)/all ITS reads × 100 at each soil sample (9).

### Network construction and visualization.

We established co-occurrence networks to identify the interactive relationships between potential phytopathogens and other trophic groups (i.e., saprotrophs and symbiotrophs) within fungal community in the field survey of agricultural soils. Fungal phylotypes (OTUs) with relative abundance more than 0.01% and occurrence in more than 20% of all soil samples were kept constructing the network. Then, the samples were separated into two groups based on the SOC content. All pairwise Spearman correlations between phylotypes were calculated using the “WGCNA” R package based on the Spearman correlation matrix (63), and *P* values were adjusted by the Benjamini and Hochberg false discovery rate (FDR) test (64). The absolute values of Spearman correlations more than 0.65 and the adjusted *P* values less than 0.01 were retained. Finally, we imported the robust correlations into the Gephi platform (version 0.9.2) (65) for network visualization using the Fruchterman Reingold algorithm.

### Statistical analysis.

One-way analysis of variance (ANOVA) was used to detect the differences in alpha-diversity (OTU richness) and relative abundance (%) of fungal phytopathogens, saprotrophs, and symbiotrophs between different fertilization treatments. Principal coordinates analysis (PCoA) based on the Bray-Curtis dissimilarity distances and permutational multivariate analysis of variance (PERMANOVA) test with 999 permutations were used to detect the differences in the beta-diversity of fungal phytopathogens, saprotrophs, and symbiotrophs in the field survey and the long-term experiment. These analyses were performed using the “vegan” package (66), and plotted using the “ggplot2” package in the R platform. To evaluate the correlations between soil properties and the diversity and relative abundance of all fungi and trophic guilds including phytopathogens, saprotrophs, and symbiotrophs, we conducted Spearman correlation analyses. A false discovery rate approach was used to determine adjusted *P* values for all the correlations to control for spurious (false positives) correlations. We used SPSS 22 (IBM, Armonk, NY, USA) to conduct these analyses. The Spearman correlations were visualized using the “pheatmap” package in the R platform. We also used the random forest model to determine the importance of each predictor on the diversity and relative abundance of all fungi and trophic guilds via evaluating the increase in the mean square error with 999 permutations, using the “rfPermute” package in the R platform. In addition, regression analyses were performed to decipher the associations between the genera of fungal phytopathogens and soil properties, and linear models were used to estimate the curve fitting and fitted with the adjusted *P* values lower than 0.05, using the “ggplot2” package in the R platform. Path analysis was performed to evaluate the effect process of the fertilization treatments and soil properties on fungal phytopathogens and their genera using SPSS AMOS 19. The fertilization treatments were categorical variables as 1 and the control as 0. The parameters including root mean square errors of approximation (RMSEA < 0.08), *χ*^2^ value (*P > *0.05), and goodness-of-fit index (GFI > 0.90) were used to indicate the model fitness.

### Data availability.

The raw sequences of fungal data sets are available in the NCBI Sequence Read Archive (SRA) database (www.ncbi.nlm.nih.gov/sra) under accession numbers PRJNA803009 and PRJNA803012 for the field survey and the long-term experiment, respectively, and the SRA accession numbers of all samples are shown in Table S5.

10.1128/msystems.01337-21.1 TABLE S1 Basic information of soil fungal phylotypes of phytopathogens, saprotrophs, and symbiotrophs in the field survey. Download Table S1, XLSX file, 0.08 MB. Copyright © 2022 Du et al. 2022 Du et al. https://creativecommons.org/licenses/by/4.0/ This content is distributed under the terms of the Creative Commons Attribution 4.0 International license.

10.1128/msystems.01337-21.2 TABLE S2 Basic information of soil properties of each sample in the field survey. Download Table S2, XLSX file, 0.02 MB. Copyright © 2022 Du et al. 2022 Du et al. https://creativecommons.org/licenses/by/4.0/ This content is distributed under the terms of the Creative Commons Attribution 4.0 International license.

10.1128/msystems.01337-21.3 TABLE S3 Basic information of soil fungal phylotypes of phytopathogens, saprotrophs, and symbiotrophs in the long-term experiment. Download Table S3, XLSX file, 0.02 MB. Copyright © 2022 Du et al. 2022 Du et al. https://creativecommons.org/licenses/by/4.0/ This content is distributed under the terms of the Creative Commons Attribution 4.0 International license.

10.1128/msystems.01337-21.4 TABLE S4 Basic information of soil properties of each sample in the long-term experiment. Download Table S4, XLSX file, 0.01 MB. Copyright © 2022 Du et al. 2022 Du et al. https://creativecommons.org/licenses/by/4.0/ This content is distributed under the terms of the Creative Commons Attribution 4.0 International license.

10.1128/msystems.01337-21.5 TABLE S5 Metadata file with SRA accessions in the field survey and the long-term experiment. Download Table S5, XLSX file, 0.02 MB. Copyright © 2022 Du et al. 2022 Du et al. https://creativecommons.org/licenses/by/4.0/ This content is distributed under the terms of the Creative Commons Attribution 4.0 International license.

10.1128/msystems.01337-21.6 FIG S1 The community structure of all fungi (a), phytopathogens (b), saprotrophs (c), and symbiotrophs (d) in the field survey. The color of each node is according to the SOC content. In b–d, the size of each node is according to the relative abundance of corresponding fungal trophic guild for each sample. Download FIG S1, EPS file, 1.7 MB. Copyright © 2022 Du et al. 2022 Du et al. https://creativecommons.org/licenses/by/4.0/ This content is distributed under the terms of the Creative Commons Attribution 4.0 International license.

10.1128/msystems.01337-21.7 FIG S2 Comparison of soil chemical properties in different fertilization treatments in the long-term experiment. The columns with asterisks in the treatments are significantly different from the control (*, *P < *0.05; **, *P < *0.01; ***, *P < *0.001). ST, crop wheat straw; CM, fresh cow manure; PM, fresh pig manure. TC, total carbon; C/N, carbon nitrogen ratio; DOC, dissolved organic carbon; DON, dissolved organic nitrogen; MBC, microbial biomass carbon; MBN, microbial biomass nitrogen. Download FIG S2, EPS file, 0.8 MB. Copyright © 2022 Du et al. 2022 Du et al. https://creativecommons.org/licenses/by/4.0/ This content is distributed under the terms of the Creative Commons Attribution 4.0 International license.

10.1128/msystems.01337-21.8 FIG S3 Comparison of fungal trophic guilds and the dominant genera in different fertilization treatments in the long-term experiment. (a and b) The diversity and relative abundance of symbiotrophic fungi in different fertilization treatments. (c) The relative abundance of the dominant genera of phytopathogenic fungi in different fertilization treatments. The columns with asterisks in the treatments are significantly different from the control (*, *P < *0.05; **, *P < *0.01; ***, *P < *0.001). ST, crop wheat straw; CM, fresh cow manure; PM, fresh pig manure. Download FIG S3, EPS file, 0.7 MB. Copyright © 2022 Du et al. 2022 Du et al. https://creativecommons.org/licenses/by/4.0/ This content is distributed under the terms of the Creative Commons Attribution 4.0 International license.

10.1128/msystems.01337-21.9 FIG S4 The community structure of all fungi (a), phytopathogens (b), saprotrophs (c), and symbiotrophs (d) in the long-term experiment. The differences between treatments and control are statistically tested by the permutational multivariate analysis of variance (PERMANOVA) approach based on Bray-Curtis dissimilarity matrices among samples. ST, crop wheat straw; CM, fresh cow manure; PM, fresh pig manure. Download FIG S4, EPS file, 1.0 MB. Copyright © 2022 Du et al. 2022 Du et al. https://creativecommons.org/licenses/by/4.0/ This content is distributed under the terms of the Creative Commons Attribution 4.0 International license.

10.1128/msystems.01337-21.10 FIG S5 Sampling map of the field survey around Fenghuang in Hunan Province and Tongren, Wanshan, and Yuping in Guizhou Province across China. Download FIG S5, EPS file, 2.5 MB. Copyright © 2022 Du et al. 2022 Du et al. https://creativecommons.org/licenses/by/4.0/ This content is distributed under the terms of the Creative Commons Attribution 4.0 International license.
